# Cognitive characteristics in women with mastectomy, mental symptoms and marriage compliance

**DOI:** 10.1192/j.eurpsy.2023.854

**Published:** 2023-07-19

**Authors:** H. B. Gürcen, S. Karaca

**Affiliations:** 1 Nurse, ÜmraniyeTraining and Research Hospital; 2 Associate Professor, Marmara University Faculty of Health Sciences Department of Psychiatric Nursing, İstanbul, Türkiye

## Abstract

**Introduction:**

Although mastectomy is a treatment for the cleaning of cancer cells in the breast, it can also cause psychological changes in women.

**Objectives:**

This research was carried out to investigate the cognitive distortions, mental symptoms, and marital adjustment relationships in women with mastectomy.

**Methods:**

The descriptive and comparatice study was conducted in a public hospital with 90 mastectomies and 90 healthy female samples. The data were collected using The Cognitive Distortions Scale (CDS), The Brief Symptom Inventory (BSI), and Marriage Adjustment Scale (MAS) and were evaluated by descriptive statistical analyzes, t test, Mann-Whitney U test and Spearman Correlation Analysis.

**Results:**

Women with mastectomy scored high in the healthy control group in all sub-dimensions of CDS and BSI and this difference is highly significant (p<0,5). Women with mastectomy scored higher in total agreement sub-dimensions than the healthy control group and the difference between them was statistically significant (p<0,5). There ara positive, very strong and statistically significant relationships between all sub-dimensions of the CDS and the total sub-dimension scores of the BSI (r=,769and, 919). There are positive, strong and statistically significant relationships between the sub-dimensions of the CDS and the sub-dimension of the agreement and the total score of MAS (r=681 and 734). There are strong and statistically significant relationships between the total and sub-dimension scores of the BSI and the sub-dimension and total score of the MAS’s agreement (r=,672 and 778).

**Conclusions:**

Cognitive distortions and mental symptoms are significantly higher in women with mastectomy, and cognitive distortions are associated with mental symptoms. Cognitive distortions and psychological symptoms scores, marriage adjustment scores and agreement sub-dimension scores increase in women.

**Disclosure of Interest:**

None Declared

